# Increasing Population Immunity Prior to Globally-Coordinated Cessation of Bivalent Oral Poliovirus Vaccine (bOPV)

**DOI:** 10.3390/pathogens13090804

**Published:** 2024-09-17

**Authors:** Nima D. Badizadegan, Steven G. F. Wassilak, Concepción F. Estívariz, Eric Wiesen, Cara C. Burns, Omotayo Bolu, Kimberly M. Thompson

**Affiliations:** 1 Kid Risk, Inc., Orlando, FL 32819, USA; 2 Global Immunization Division, Global Health Center, Centers for Disease Control and Prevention, Atlanta, GA 30333, USA; 3 National Center for Immunization and Respiratory Diseases, Centers for Disease Control and Prevention, Atlanta, GA 30333, USA

**Keywords:** polio, eradication, dynamic modeling, OPV cessation, immunization

## Abstract

In 2022, global poliovirus modeling suggested that coordinated cessation of bivalent oral poliovirus vaccine (bOPV, containing Sabin-strain types 1 and 3) in 2027 would likely increase the risks of outbreaks and expected paralytic cases caused by circulating vaccine-derived polioviruses (cVDPVs), particularly type 1. The analysis did not include the implementation of planned, preventive supplemental immunization activities (pSIAs) with bOPV to achieve and maintain higher population immunity for types 1 and 3 prior to bOPV cessation. We reviewed prior published OPV cessation modeling studies to support bOPV cessation planning. We applied an integrated global poliovirus transmission and OPV evolution model after updating assumptions to reflect the epidemiology, immunization, and polio eradication plans through the end of 2023. We explored the effects of bOPV cessation in 2027 with and without additional bOPV pSIAs prior to 2027. Increasing population immunity for types 1 and 3 with bOPV pSIAs (i.e., intensification) could substantially reduce the expected global risks of experiencing cVDPV outbreaks and the number of expected polio cases both before and after bOPV cessation. We identified the need for substantial increases in overall bOPV coverage prior to bOPV cessation to achieve a high probability of successful bOPV cessation.

## 1. Introduction

Well past the year 2000 target date set for poliomyelitis (polio) eradication in 1988, all three types of polioviruses (i.e., types 1, 2, and 3) continued to cause cases of paralytic polio [1], with ongoing transmission of some polioviruses reported in 2024 [2,3]. Historically, nearly all countries used oral poliovirus vaccine (OPV) in a trivalent formulation (tOPV, containing all three types) to achieve high levels of population immunity and stop the indigenous transmission of all wild polioviruses (WPVs). Countries that did not achieve and maintain sufficiently high routine immunization (RI) coverage to stop and prevent WPV transmission conducted supplemental immunization activities (SIAs) either as planned, preventive SIAs (pSIAs) or reactive, outbreak response SIAs (oSIAs) [1].

Before 2000, the potential of eradication dividends similar to smallpox eradication [4] and recognition of OPV-related risks supported discussions about ending poliovirus vaccine use after successful polio eradication, particularly OPV [1,5]. Specifically, after achieving high national immunization coverage with tOPV and eliminating indigenous WPV transmission, some high-income countries began to recognize the small but non-zero risks of OPV. First, OPV causes rare cases of vaccine-associated paralytic polio (VAPP) in vaccine recipients and their close contacts, which means a small number of expected polio cases each year as long as OPV use continues [1,5,6]. Second, low overall immunization coverage in OPV-using countries allows sustained transmission of OPV-related viruses among under-vaccinated individuals that can lead to the emergence of vaccine-derived polioviruses (VDPVs), which cause outbreaks of circulating VDPVs (cVDPV, recently also called variant polioviruses [3]) that behave identically to homotypic WPVs [6]. Last, prolonged OPV infections in some individuals with some primary immunodeficiencies can lead to very rare long-term shedding and paralytic cases due to immunodeficiency-associated VDPVs (iVDPVs) [6]. In 2008, the World Health Assembly (WHA) resolved to develop a plan “to set, if and when appropriate, a date for the eventual cessation” of OPV use in RI after the successful eradication of WPVs [7]. A 2008 health economic analysis noted that opportunities to enhance population immunity prior to OPV cessation could substantially reduce the expected burden of paralytic cases after cessation by achieving maximum population immunity, particularly in low-income countries [8].

As the Global Polio Eradication Initiative (GPEI) experienced delays in achieving polio eradication milestones, it supported the development and introduction of additional OPV formulations [1]. GPEI proposed using monovalent OPV (mOPV), and later bivalent OPV (bOPV, Sabin-strains type 1 and 3), in some SIAs in the most challenging poliovirus transmission areas to overcome relative “vaccine failure” caused by interference of type 2 in tOPV with the immune response to types 1 and 3 [1,9]. The use of Sabin-strain mOPV formulations for types 1 (mOPV1) and 3 (mOPV3) began in some SIAs in the mid-2000s, and the use of bOPV (containing types 1 and 3) started in 2009 [1]. However, any advantages of slightly increased per-dose immunogenicity for the single OPV type in mOPV (or two types in bOPV) became negligible with the administration of multiple OPV doses to the same populations in successive SIA rounds [1]. Ultimately, this strategy opened up immunity gaps for the OPV types not included in these SIAs, which allowed a resurgence of WPV3 cases (following the use of mOPV1), and vice versa and increased the risks of type 2 cVDPV (cVDPV2) emergence [10,11,12,13,14,15]. After 2009, the number of reported cVDPV2 cases increased substantially in areas using tOPV with low RI coverage and insufficient tOPV SIAs [3,16], which, along with ongoing risks of VAPP and iVDPV, led GPEI to shift to a policy of phasing OPV cessation by poliovirus type, starting with type 2 [17]. Preparations for type 2 OPV (OPV2) cessation included conducting pSIAs with tOPV to increase immunity to type 2 in populations with immunity gaps prior to April 2016. In April-May 2016, GPEI globally coordinated the cessation of all use of OPV2 for preventive immunization (i.e., RI and pSIAs), and all countries using tOPV in RI replaced it with bOPV and added at least one dose of inactivated poliovirus vaccine (IPV) into their RI schedules [17,18]. After May 2016, OPV2 became restricted to use for oSIAs with vaccine only available from a global stockpile of Sabin-strain type 2 mOPV (mOPV2) controlled by the World Health Organization (WHO) [17,18,19,20,21].

### 1.1. bOPV Cessation Planning Prior to OPV2 Cessation

At the time of OPV2 cessation, discussions among GPEI partners about the high number of cVDPV2s prior to 2015 led to the recognition of the importance of continuing to implement preventive bOPV pSIAs until bOPV cessation to ensure high population immunity for types 1 and 3. Considering the results of modeling [22,23], in October 2016, the WHO Strategic Advisory Group of Experts (SAGE) polio workgroup concluded that:

“If the current level of routine bOPV and IPV coverage is maintained, most countries will not require additional bOPV campaigns prior to OPV cessation. However, if bOPV SIAs are not maintained and population immunity drops prior to OPV cessation, then areas with high force of infection and low RI coverage (especially in areas with under-vaccinated and/or inaccessible sub-populations), will need to conduct multiple bOPV campaigns prior to bOPV cessation to prevent cVDPVs after bOPV cessation”.

[24]

The workgroup further recommended maintaining the following:

“ongoing preventive SIAs in countries with low routine immunization coverage and additional bOPV campaigns prior to OPV cessation in countries (areas) where population immunity remains low”.

[24]

SAGE formally adopted these recommendations in 2016 [25]. GPEI strategic plans and budgets up through 2019 (with bOPV cessation anticipated to occur in 2019 [26]) included maintenance of bOPV pSIAs to sustain high levels of population immunity and mitigate the risks of outbreaks from WPV (or cVDPV) importations as well as cVDPV emergence for types 1 and 3 prior to and after OPV2 cessation [1].

### 1.2. bOPV Cessation PlanningFollowing OPV2 Cessation

Multiple emergences and widespread transmission of cVDPV2s after 2018 [27,28,29] led GPEI to shift resources budgeted for bOPV pSIAs to mOPV2 oSIAs [1,19]. GPEI also reinstated the production of tOPV for the OPV2 stockpile using existing Sabin OPV2 bulk for use in areas with co-circulation of different types of polioviruses [14,19,21,30]. Following research and development efforts initiated in 2011 that sought to genetically modify Sabin OPV strains to reduce their potential for reversion to cVDPVs, in 2019, GPEI accelerated the development of a type 2 novel OPV (nOPV2) and commissioned nOPV2 doses for the OPV2 stockpile [19,31,32,33]. In 2020, GPEI plans anticipated sufficient nOPV2 supply by early 2021 [34], and SAGE recommended preferential use of nOPV2 (see Section 5.4 of [35,36]), with countries in the WHO African Region opting to delay oSIAs to wait for nOPV2 instead of using Sabin-strain mOPV2. Additionally, disruptions due to the COVID-19 pandemic led to the cancellation or postponement of many oSIAs in 2020. In November 2020, nOPV2 received an emergency use listing (EUL) from WHO, and in December 2023, nOPV2 became fully licensed.

Following the GPEI shift of resources from bOPV pSIAs to OPV2 oSIAs [1], the number of cVDPV1 reported cases increased. Co-circulation of poliovirus type 1 (i.e., WPV1, cVDPV1) and 2 (i.e., cVDPV2) also emerged as an increasing challenge. During 2021–2023, GPEI reported more annual cases caused by type 1 cVDPVs (cVDPV1) than those caused by WPV1 [1,2,3]. In October 2020, SAGE recommended that “tOPV be made available to countries for cVDPV2 outbreak response in subnational areas where there is co-circulation or high risk of co-circulation of cVDPV2 with cVDPV1, cVDPV3 or WPV1 in order to avoid the need to conduct dual mOPV2 and bOPV campaigns” [36]. Several countries used tOPV from the OPV2 stockpile during 2021–2023 to control cVDPV2 outbreaks that occurred contemporaneously with WPV1 transmission (i.e., Afghanistan, Pakistan), cVDPV1 (i.e., Yemen) or due to high-risk of co-circulation (i.e., prior experience with a cVDPV3 outbreak in 2018 in Somalia in 2018 and ongoing concerns about risks of cVDPVs there).

Ongoing poliovirus transmission in 2024 [2,3] indicated missed milestones of the GPEI 2022–2026 strategic plan, which aimed to end all WPV1 and cVDPV transmission by the end of 2023 ([1], ([37], see page xi). Nonetheless, bOPV cessation planning discussions continue since the plan suggested that bOPV cessation could occur as early as 2027, with implementation plans developed at least two years before bOPV cessation [37]. With respect to SIAs, the plan stated: “SIAs with types 1 and 3 containing OPV (Sabin or novel) should be implemented over a period of time in the years prior to cessation and not just during the immediate pre-cessation period, to maintain persistently high population-level immunity” ([37], see page 42). This has not occurred.

Polio modeling studies continue to provide analytical support for deliberations by GPEI partners [38,39,40,41,42] (also see reviews of polio modeling studies published 2000–2019 [43] and 2020–2024 [44]). Recognizing that modeling studies may help with bOPV planning efforts and building on our prior studies related to bOPV cessation [22,45,46,47,48,49], this study aims to simulate the potential benefits of intensifying bOPV pSIAs years prior to bOPV cessation. The next section provides a review of our prior global OPV cessation modeling publications for readers unfamiliar with polio modeling. Section 3, Section 4 and Section 5 present the methods, results, and discussion of our updated modeling of bOPV cessation with and without the addition of bOPV pSIAs.

## 2. Global Poliovirus Transmission Modeling

### 2.1. Characterization of OPV Risks and Global Modeling Concepts

Prior to any decisions about OPV cessation, modeling studies identified potential variability in national preferences for vaccine options after polio eradication (i.e., IPV, OPV, or no vaccine) [8,50], the importance of global coordination of OPV cessation and creation of an OPV stockpile to support rapid oSIAs if needed [20,46,51,52,53], and prerequisites for OPV cessation that would help manage risks [54]. Our modeling emphasized the importance of increasing population immunity [10], which we define for the entire population relative to stopping poliovirus transmission, in contrast to other definitions of vaccine-induced immune protection from paralysis in children under a specific age (e.g., age 3 years old) [43,44]. Multiple studies identified the need to intensify tOPV pSIAs prior to OPV2 cessation to reduce the risks of emergence of cVDPV2 outbreaks after OPV cessation [14,23,55] and demonstrated the limited role of IPV in post-OPV2 cessation risk management [56]. Several studies highlighted the importance of rapidly detecting any ongoing cVDPV2 transmission after OPV2 cessation to promptly and aggressively conduct OPV2 oSIAs with high coverage to stop transmission [21,23,57]. Global modeling studies identified a small chance of failure of OPV cessation as a polio endgame strategy and the potential need to restart tOPV production and preventive use (i.e., OPV restart) if OPV cessation did not succeed [21,23].

We developed and maintained an integrated global poliovirus transmission, risk, decision, and economic model to explore the health and economic risks, costs, and benefits of polio endgame strategies (for full model description, assumptions, inputs, and equations, see [58] and its associated technical appendix). Briefly, our integrated global polio models compartmentalize the world population into subpopulations that simulate dynamic demographics, immunization activities, epidemiological histories, and characteristics relevant to poliovirus transmission, with stratifications required to characterize variability consistent with differences in actual conditions recognized through experience. For each subpopulation, which includes multiple age groups, we apply a deterministic transmission model that includes different immunity states accounting for each of the 3 different poliovirus types to simulate immunity induced by maternal antibodies, different combinations of OPV and/or IPV doses from immunization, prior infections, and waning of immunity [11]. Notably, the transmission model includes the potential for individuals with prior immunity to potentially become reinfected and participate in transmission, although only fully susceptible individuals may develop paralysis with poliovirus type-specific rates [11]. The transmission model includes a multistage infection process, and mixing occurs within model subpopulations heterogeneously by age and homogeneously in space and between subpopulations according to varied preferential mixing areas that represent larger geographical regions [11,58]. The model also includes a multistage OPV evolution process to simulate the loss of attenuating mutations and increases that occur in fitness, transmissibility, and potential for neurovirulence of OPV and OPV-related viruses in populations to mimic the emergence of cVDPVs, with infections of fully susceptible individuals with an OPV or OPV-related virus able to lead to paralysis with type- and OPV-evolution stage-specific rates [11,16]. Given the complexity of the different types and formulations of poliovirus vaccines, the model includes full consideration of their properties and appropriate adjustments for differences between populations (e.g., relatively lower or higher take rates in lower-income, temperate, or higher-income countries based on evidence from vaccine effectiveness studies) [58].

Our integrated global model introduced more complexity over time, and assumptions were modified as global conditions changed [23,28,48,49,58,59,60,61,62,63]. For all applications, the model includes a historical “burn in” period that includes retrospective deterministic inputs based on the available evidence (i.e., immunization coverage and epidemiological data) to generate appropriate initial conditions. Then, for prospective analyses, the integrated model includes numerous iterations to account for uncertain potential stochastic risk events (e.g., reintroduction from importation, containment breach, iVDPVs) and considers different potential mixing between subpopulations. Stochastic simulations yield numerous possible future realizations of the world for the different scenarios and/or policies considered, which we typically communicate in the form of expected values that average over all of the stochastic iterations and/or cumulative outcomes (e.g., cases, costs, probability of successful die-out) for the prospective model time horizon.

### 2.2. OPV Cessation Global Modeling Pre-OPV2 Cessation

Global modeling of the polio endgame trajectory prior to OPV2 cessation assumed that GPEI and countries would follow model- and SAGE-recommended risk management strategies to achieve the best chances of success [21,23]. These recommendations included pre-OPV2 cessation tOPV intensification, maintenance of bOPV pSIAs until bOPV cessation, sufficient and aggressive oSIAs to promptly stop outbreaks using homotypic OPV for 5 years after OPV cessation, then use IPV [23], with a separate exploration allowing homotypic OPV use for the entire model time horizon (e.g., through 2052) [21]. Additional analyses performed a larger number of stochastic iterations to identify more potential root causes for endgame failure [64] and to explore the implications of using OPV for 0 to 5 years before using IPV for oSIAs and of not conducting any oSIAs for type 2 poliovirus outbreaks after 2016 [65]. However, recognizing some chance of failure of OPV2 cessation and later bOPV cessation, the model included a threshold of 50,000 total cases (sensitivity analysis range of 1000 to 50,000) of all poliovirus types combined since OPV cessation to trigger OPV restart (i.e., tOPV use in RI and/or pSIAs) [23,29]. The pre-OPV2 cessation modeling studies assumed initial conditions as of the end of 2014 and led to an estimated 2 OPV restarts in 100 stochastic model iterations [23] and 57 OPV restarts out of 1000 stochastic iterations [64] for 2013–2052, with sensitivity analyses and different assumptions about OPV use in oSIAs leading to more expected failures and thus more OPV restarts [21,23,64,65]. Additional modeling characterized the expected increase in vulnerability of populations to restarted poliovirus transmission and outbreaks as a function of time after OPV cessation and the type of poliovirus imported [66,67].

For OPV types 1 and 3, pre-bOPV cessation modeling studies characterized (i) the expected benefit of maintaining high population immunity for types 1 and 3 with bOPV pSIAs until bOPV cessation and (ii) the expected increase in vulnerability of populations to restarted poliovirus transmission and outbreaks as a function of time after OPV cessation and type of poliovirus imported [22,45]. With the last reported case caused by WPV3 reported in 2012, studies also considered the implications of globally coordinated cessation of type 3 OPV (OPV3) prior to type 1 OPV (OPV1) [46,47].

### 2.3. OPV Cessation Global Modeling Post-OPV2 Cessation

After OPV2 cessation (i.e., since 2016), continued cVDPV2 transmission and delayed achievement of WPV1 eradication increased the complexity of polio endgame modeling, particularly with respect to the potential for OPV restart, bOPV cessation, increasing poliovirus vaccine options [46,68,69]. Following the release of the GPEI 2019–2023 Strategic Plan, we updated the global model to reflect the actual initial conditions as of the end of 2019 and shifted the global model time horizon to 2019–2029. At that time, we changed the OPV restart threshold to three type-specific limits of 5000 cases since homotypic OPV cessation [58]. We also updated the model inputs to include: (i) oSIAs with suboptimal performance qualities as observed since OPV2 cessation, (ii) mOPV2 use allowed for oSIAs up to 8 years after OPV2 cessation (i.e., 2024), or alternatively for the entire model time horizon, (iii) bOPV cessation in 2025 (or alternatively after the end of the model time horizon) with homotypic mOPV use for oSIAs allowed for types 1 and 3 for 5 years after bOPV cessation, and (iv) updated risk estimates for reintroduction from unexpected use of OPV after OPV cessation [28]. As efforts to develop nOPV2 accelerated, we updated the model to include bounding cases for its characteristics, and we explored the impact of using nOPV2 for oSIAs instead of mOPV2 [59]. A health economic analysis explored different options for RI vaccines assuming different scenarios for control (i.e., not pursuing bOPV cessation and either restarting tOPV or switching to 2 doses of IPV) and an eradication scenario (i.e., bOPV cessation in 2025 using modeled SIA inputs that led to successful WPV1 eradication by 2023) [70]. These studies published in 2020–2021 reported increased expected numbers of prospective stochastic model iterations with OPV restarts [28,59,70].

Following the emergence of SARS-CoV-2 and the COVID-19 pandemic, we updated the global model assumptions for long-range exportation risks and explored the impacts of disruptions in immunization and transmission due to reduced mixing during the initial stages of the pandemic, again considering mOPV2 and nOPV2 options for oSIAs [60]. This model used the same initial conditions (through the end of 2019) and a model time horizon of 2019–2023 [60]. A subsequent study focused on oSIAs with the same time horizon of 2019–2023 but started with initial conditions updated to the end of 2020 and explored the consequences of delaying oSIAs to wait for nOPV2 instead of using mOPV2 [61]. Another study using a time horizon of 2022–2026 with initial conditions updated to the end of 2021 explored the trade-offs for numerous oSIA performance characteristics (including the identification of some aggressive global strategies with more rounds, larger rounds, expanded age target age groups, improved coverage per round) and vaccine options [62]. These studies with shorter (i.e., 4-year) time horizons reported the potential for fewer expected OPV2 restarts depending on oSIA vaccine options and performance characteristics [60,61,62].

In 2019, GPEI did not restart the preventive use of OPV2 (i.e., in RI or pSIAs) when it commissioned new bulk production of nOPV2 for the global stockpile [71]. Following the introduction of nOPV2, GPEI shifted its outbreak response expectations to include ongoing production and exclusive use of nOPV2 for oSIAs [72] with no option for OPV restart. Consequently, since 2022, our global modeling studies have not included the possibility of OPV restarting for preventive use. Instead, we allowed OPV use for oSIAs for the entire model time horizon and focused on characterizing the probability of die-out of transmission by the end of the model time horizon [29,48,49,73]. Using a model time horizon of 2022–2026 or 2022–2035, and based on the contemporary experience of suboptimal oSIA success in stopping outbreaks, these studies reported <1% chance of dying out of type 2 transmission by the end of the model time horizon [29,48,49,59]. These studies also highlighted increased global risks of co-circulation of types 1 and 2 and the potential for substantial reductions in expected costs and cases with the use of tOPV if available or co-administered nOPV2 and bOPV in oSIAs instead of sequential oSIAs for different OPV types [29,48,49,59].

With respect to bOPV cessation, the global modeling conducted in 2022 explored the implications of projected GPEI and country plans as of the end of 2021 and assumed bOPV cessation around 1 May 2027 [48,49]. These studies reported numerous expected cVDPV1 cases both before and after bOPV cessation in the absence of bOPV pSIAs and reported a <1% chance of the die-out of type 1 transmission by the end of the model time horizon [48,49].

### 2.4. Characterizing the Probability of Success (POS) for OPV Cessation

To harmonize across the use of different metrics for OPV cessation performance in the various models described above, we broadly characterize model estimates of the probability of success (POS) for ending poliovirus transmission after OPV cessation using either (i) 1 minus the probability of OPV restart or (ii) the probability of die-out. Table 1 summarizes the evolution of published estimates with quantitative POS estimates for OPV cessation and key attributes of the studies (e.g., metric reported, model time horizon and initial conditions, poliovirus types included in the OPV restart criteria, the POS estimate(s), publication date and source) [21,23,28,48,49,59,60,61,62,64,65,70,73]. The duration of the model time horizons shortens considerably from top to bottom in Table 1, which reveals decreasing POS estimates over time while also allowing for fewer numbers of years to observe failure (i.e., increased right-censoring of the results). Pre-OPV2 cessation studies implied a high expected POS (i.e., >90% for all OPV types combined), while the expected POS for type 2 dropped to 11% for a 10-year time horizon post-OPV2 cessation [28,59,70]. Later studies with shorter (i.e., 4-year) time horizons reported potentially higher and variable type 2 POS estimates depending on oSIA vaccine options and performance characteristics [60,61,62], but modeling since 2022 reported expected POS results of <1% for types 1, 2, and 3, assuming GPEI continues current policies and program performance remains the same.

Additional retrospective analyses identified differences between pre-OPV2 cessation model assumptions and post-OPV2 cessation experience [58,74]. A 2023 look back concluded that the authors knew then what they know now about the implementation of pre-OPV2 cessation activities and post-OPV2 cessation oSIAs; pre-OPV2 cessation modeling would not have supported expectations of a high POS for OPV2 cessation in 2016 [29]. The decline in POS estimates primarily reflects the overall failure to fully implement pre-OPV2 cessation pSIAs and model-recommended oSIA timeliness, scope, number, and quality [21]. This serves as a powerful reminder that pre-OPV cessation immunization activities, while essential for success if implemented well, do not represent the only critical factor for a successful polio endgame.

## 3. Materials and Methods

For this study, we started with the 2023 model of bOPV cessation without additional bOPV pSIAs (i.e., very limited use of bOPV SIAs only in some countries and no planned bOPV intensification) [48] to explore the role of bOPV pSIA intensification prior to OPV cessation. The current formulation of the global model divides the world into 72 blocks of 10 subpopulations (i.e., 720 subpopulations) of approximately 10.7 million people each, stratified by current RI vaccine use (i.e., OPV + IPV, IPV/OPV, IPV-only) and World Bank Income Level (low-income, LI; lower-middle-income, LMI; upper-middle-income, UMI; high-income, HI). The model assumed historical RI coverage consistent with WHO/UNICEF estimates, with some transient adjustments for the disruptions associated with the COVID-19 pandemic [48]. We assume SIA performance characteristics related to coverage and repeatedly missing the same children by successive SIA rounds as in prior modeling [48]. The model detects any cases of any poliovirus type at the time of onset of paralysis, which does not account for additional delays that may exist in practice [75], and assumes that oSIAs start 45 days after detection in the model, which accounts for some delay for surveillance. All oSIAs target children < 5 years of age and include 2 oSIA rounds 30 days apart, with one recent exception noted below. The model automatically implements 2 additional rounds after any breakthrough transmission, for which we assume the oSIA occurs only in the outbreak subpopulation if its basic reproductive number (R_0_) for type 1 WPV (WPV1) < 10. For other subpopulations with WPV1 R_0_ ≥ 10, oSIA rounds occur in the outbreak subpopulation and its four worst-performing neighbor subpopulations within the same block. We vary the oSIA intensity for different subpopulations, which ranges from 15% true coverage and 95% repeatedly missed probability to 80% true coverage and 50% repeatedly missed probability. We also assume the use of Sabin OPV for oSIAs prior to type-specific OPV cessation and model different OPV vaccine options after type-specific OPV cessation. When triggered in the model, oSIAs replace or delay any pSIA rounds, similar to the GPEI experience [28,48,49,58,59,60,61,62,63]. For this analysis, we assume the use of Sabin mOPV2 occurred from 2016–2021 and the use of nOPV2 from 2022 on. Similar to prior modeling, we apply bounding scenarios of (i) nOPV best, which uses type-specific nOPV for outbreak response assuming the same effectiveness as type-specific mOPV, no reversion despite transmissibility, and no VAPP, and (ii) nOPV worst, which uses type-specific nOPV for outbreak response post-type-specific OPV cessation, assumes 90% of the effectiveness of mOPV and prior assumptions for reduced reversion [59], which we further reduced here by 10%, and VAPP occurring at a rate 10% lower than the VAPP rate of mOPV in vaccine recipients [48].

Recognizing accelerated efforts to develop nOPV for types 1 and 3 (i.e., nOPV1 and nOPV3), for this analysis, we assume the availability of these vaccines for oSIAs at the time of bOPV cessation, starting 1 May 2027. Thus, we consider the implications of using nOPV2 starting at the beginning of the time horizon (in 2022) for the nOPV scenarios, with bOPV used for type 1 and/or 3 oSIAs prior to 2027 and nOPV1 and nOPV3 use after bOPV cessation. We focus on exploring the relative benefits of resuming and increasing bOPV pSIAs prior to bOPV cessation as a strategy to reduce expected cVDPV cases before and after bOPV cessation.

Since 2023 modeling [48] included implementation of some bOPV pSIAs prospectively in years 2022 and 2023 that did not occur, and it did not include some oSIAs in those years that did occur, we adjusted these baseline SIA inputs appropriately and shifted the model time horizon to 2024–2035. We also identified the very low SIA coverage in the model blocks that represent the conditions in countries such as Nigeria, the Democratic Republic of the Congo (DRC), and Yemen as barriers to success, noting that GPEI partners have invested substantial resources in these consequential geographies since 2022. This led us to assume an increase in the SIA quality in Nigeria and DRC starting in 2022 and 2023, respectively, such that for these blocks, we returned SIA quality to the pre-COVID levels (i.e., we raised the *SI_L_* model input by one step for each). We also updated the baseline to include the use of tOPV in more SIAs than in prior modeling [48] since these SIAs actually occurred in Pakistan, Afghanistan, and Yemen, and we increased the target age range for oSIAs in Yemen to children < 10 years for consistency with reported implementation [76]. In addition, given the continuing transmission of WPV1, we postponed the assumed timing of post-eradication degradation of surveillance quality in prior global modeling [48] from 2025 to the 2035 end of the model time horizon. Maintaining the quality of surveillance significantly changed the expected numbers of polio cases after 2027 compared with prior modeling [48] because it allows for earlier outbreak detection and earlier performance of oSIAs. While we modeled these changes here, uncertainty remains about GPEI plans, finances, and potential future dissolution. Notably, GPEI already reports insufficient resources to support bOPV pSIAs due to the substantial resource needs for oSIAs [1,77]. Given these changes, we reran the baseline we used for the prior study (with no additional bOPV pSIAs [48]) for the nOPV scenarios (i.e., nOPV best and nOPV worst). We do not include an updated mOPV baseline because we assume a global shift to nOPV2 oSIA responses since 2022.

**Figure 1:**
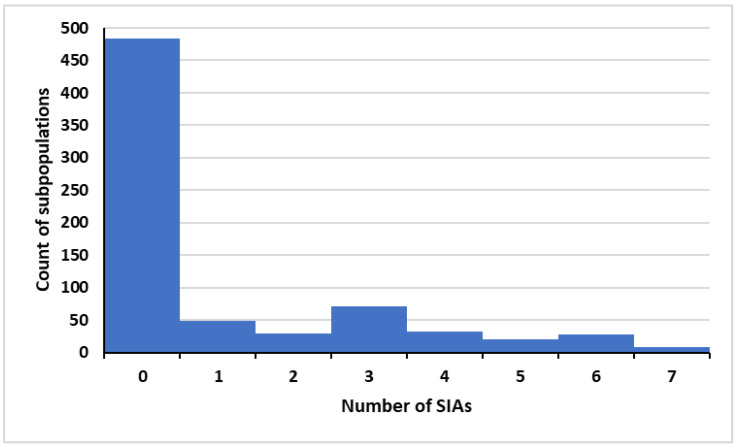
Histogram of bOPV pSIAs in the 720 model subpopulations with intensification *. Abbreviations: bOPV, bivalent OPV, pSIA, preventive supplemental immunization activity. * Pakistan and Afghanistan 6 pSIAs (low coverage areas 7 pSIAs), most of the Democratic Republic of the Congo, Nigeria, and Ethiopia 2–3 pSIAs (low coverage areas 4–7 pSIAs), most of India 3 pSIAs (high-risk areas, including UP and Bihar, 5–7 pSIAs), most of Somalia and South Sudan 2 pSIAs (low-coverage areas 7 pSIAs), most of Ukraine 4 pSIAs (high-risk areas 6 pSIAs), Yemen and Papua New Guinea 6 pSIAs, most of Indonesia 1 pSIA (low coverage areas 3–4 pSIAs), most of the Syrian Arab Republic 1 pSIA (low coverage areas 2–3 pSIAs), most of Bangladesh 3 pSIAs (low coverage areas 5 pSIAs), Côte d’Ivoire, Mauritania, Egypt, and Haiti 4 pSIAs, Philippines 3 pSIAs, 3–4 pSIAs in low-coverage areas in modeled subpopulations that include countries such as: Albania, Algeria, Angola, Armenia, Azerbaijan, Benin, Bosnia and Herzegovina, Botswana, Burkina Faso, Burundi, Cambodia, Cameroon, Central African Republic, Chad, Comoros, Congo, Djibouti, Dominican Republic, El Salvador, Equatorial Guinea, Eritrea, Eswatini, Ethiopia, Gabon, Gambia, Georgia, Ghana, Guatemala, Guinea, Guinea-Bissau, Haiti, Honduras, Kazakhstan, Kyrgyzstan, Kenya, Lao People’s Democratic Republic, Lesotho, Liberia, Libya, Madagascar, Malawi, Mali, Mauritius, Montenegro, Morocco, Mozambique, Myanmar, Namibia, Nicaragua, Niger, Papua New Guinea, Philippines, Republic of Moldova, Rwanda, Senegal, Serbia, Sierra Leone, Somalia, State of Palestine, Sudan, Tajikistan, The Former Yugoslavian Republic of Macedonia, Togo, Tunisia, Turkmenistan, Uganda, Ukraine, United Republic of Tanzania, Viet Nam, Zambia, and Zimbabwe. The global model includes some of these countries due to risks posed by other countries in the same block. Generally, countries with WPV1 R_0_ ≥ 10 with any levels of coverage would likely benefit from some pSIAs (e.g., the inclusion of pSIAs in India and Bangladesh), and all countries with subpopulations with coverage less than <60% would likely need 3–4 pSIAs. The model does not provide refined estimates of the number of pSIAs and may not fully account for differential decreases in coverage that occurred during COVID-19 and persist in some countries.

Starting with the updated baselines, we also consider the potential impact of resuming and/or increasing bOPV pSIAs in the subpopulations in the model characterized by low RI coverage and high transmission risks that correspond to countries that historically performed SIAs. We focused on increasing the number of annual bOPV pSIA rounds starting in the early part of each calendar year from 2025 through the implementation of bOPV cessation in the model on 1 May 2027. We used an iterative process to identify the specific model subpopulations that would benefit from bOPV pSIAs and sought to include enough rounds to potentially stop all ongoing cVDPV1 transmission by bOPV cessation in 2027. For 2024, we included 6–7 bOPV pSIAs in 2024 for Pakistan and Afghanistan and 2 bOPV pSIAs for high-risk areas in northern India. Figure 1 shows a histogram of the number of added bOPV pSIAs for the 720 model subpopulations for 2025 and 2026. Increasing the number of pSIAs implicitly creates more opportunity for both bOPV pSIAs and any needed type 2 oSIAs to occur. All 20 subpopulations in the model that represent populations of the endemic countries (Pakistan and Afghanistan) perform between 3 and 7 bOPV pSIAs per year, with the higher number representing implementation of SIAs in the most challenging areas. For the non-endemic countries, the note in Figure 1 provides context about the subpopulation characteristics that motivated the addition of different numbers of bOPV pSIAs.

As in prior prospective analyses, we include stochastic risks of reintroduction of polioviruses from outbreaks into other subpopulations as well as random events (e.g., post-cessation unexpected use of OPV, excretion into the community of immunodeficiency-associated VDPVs, and/or containment breaches). We present the results of the model simulations using the expected value of 100 stochastic iterations, performed using JAVA^TM^ programming language in the integrated development environment Eclipse^TM^, starting with the same random number seeds and initial conditions to control the stochasticity for each scenario to focus on direct comparisons of the different scenarios.

## 4. Results

**Figure 2:**
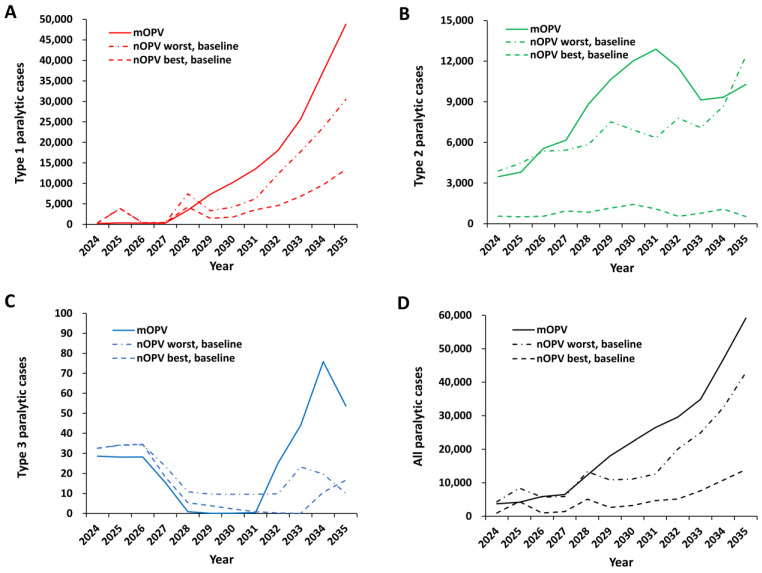
Expected annual polio cases for global bivalent oral poliovirus vaccine (bOPV) cessation occurring in 2027 and outbreak response for using monovalent OPV (mOPV) (assumptions and result from the prior study [48]), or using updated assumptions for novel OPV (nOPV). Outbreak response scenarios for nOPV (baseline) assume nOPV2 best or nOPV2 worst from 2022 on, and outbreak response for type 1 or 3 using Sabin-strain bOPV until 2027, and then either homotypic nOPV best or nOPV worst for types 1 and 3 (see text and prior studies [48,49] for assumed characteristics of nOPV best and nOPV worst bounds).

Figure 2 shows the expected values of the paralytic polio cases for the model time horizon for the baseline from the prior study (i.e., mOPV) [48] and for the new baselines (i.e., nOPV best and nOPV worst). The prior baseline assumed some continued bOPV pSIAs that did not actually occur and assumed mOPV2 use for oSIAs for the full-time horizon [48]. As shown in Figure 2, the baseline expected total cases for types 1 and 2 for the nOPV best and nOPV worst scenarios reflect: (i) the removal of previously modeled bOPV pSIAs for 2022 and 2023 [48], which did not actually occur, (ii) the inclusion of some tOPV and mOPV2 oSIAs that did occur, and (iii) the shift to nOPV2 for most oSIAs in 2022 and 2023. Figure 2A shows a somewhat higher overall expected burden of type 1 cases from 2024–2027, although these remain relatively low as long as bOPV use continues in RI and oSIAs. This occurs because the model includes oSIAs with bOPV for any type 1 outbreak prior to bOPV cessation, in addition to its use in RI and any pSIAs included for 2022–2027 in the polio-endemic countries of Pakistan and Afghanistan. Similar to prior modeling [48], the implementation of bOPV cessation in 2027 without additional bOPV pSIAs shows a rapid and substantial increase in expected type 1 paralytic polio cases. Figure 2B shows the expected number of annual type 2 cases. Since the introduction of nOPV2 starts in 2022, the expected cases for type 2 with oSIAs that use *mOPV*, nOPV best, or nOPV worst already differ in 2024. Figure 2B shows a much lower expected number of type 2 cases if nOPV2 exhibits unrealized properties similar to those modeled for the *nOPV2 best* bound, but the expected cases do not go to zero. The low expected number of type 3 paralytic cases shown in Figure 2C reflects the outcome of a relatively small fraction of stochastic iterations with any type 3 poliovirus transmission. Combining the expected cases for the scenarios in Figure 2D shows the dominance of the expected type 1 cases due to its relatively greater transmissibility and neurovirulence. Notably, the baseline scenario leads to increasing use of bOPV oSIAs to manage cVDPV1 outbreaks, and in some subpopulations and iterations, co-circulation of cVDPV1 and cVDPV2 outbreaks that the model manages by prioritizing oSIAs to the first outbreak type observed. The results in Figure 2 continue to reflect the model assumptions related to limited to no improvements in oSIAs and no restart of any preventive OPV use, independent of the burden of disease (i.e., no consideration of reactive policy changes).

**Figure 3:**
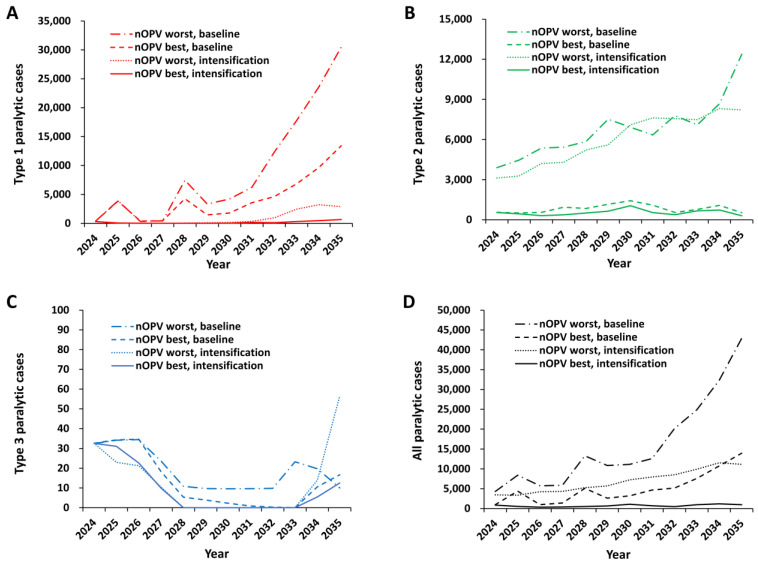
Expected annual polio cases for global bivalent OPV (bOPV) cessation in 2027 and outbreak response for type 2 using novel OPV (nOPV) with either nOPV2 best or nOPV2 worst from 2022 on, and outbreak response for type 1 or 3 using Sabin-strain bOPV until 2027, and then either homotypic nOPV best or nOPV worst (see text and prior studies [48,49] for assumed characteristics of nOPV best and nOPV worst bounds) after bOPV cessation without (baseline) and with additional preventive supplemental immunization activities using bOPV (i.e., intensification) added in some model subpopulations (see text and Figure 1).

Figure 3 shows the expected annual cases with additional bOPV pSIAs (i.e., intensification) in some model subpopulations to intensify population immunity between 1 January 2024 and 1 May 2027, see Figure 1) compared with the baseline. In contrast with prior modeling [48] and the new baselines (Figure 2), the addition of bOPV pSIAs leads to a substantial reduction in the number of expected cases for type 1, as shown in Figure 3A for the entire time horizon. Overall, the expected number of annual cVDPV1 cases remains relatively low as long as bOPV use continues, but bOPV cessation in 2027 increases the risks of cVDPV1 outbreaks and expected cases after 2027. Due to the change in assumptions about surveillance and oSIA coverage in some high-risk geographies, the results show a less dramatic expected increase in cVDPV1 cases than prior results [48]. As expected, Figure 3B shows that adding bOPV pSIAs does not substantially change the expected annual cases for type 2, with the nOPV best and nOPV worst curves showing similar annual expected cases, although some differences occur due to some iterations with co-circulation. Adding bOPV pSIAs lowers the number of iterations with type 3 transmission and expected cases, and Figure 3C shows similar low expected annual cases compared with Figure 2C. A comparison of the total expected annual cases for the scenarios in Figure 3D shows a substantial improvement over the expected results in Figure 2D.

Table 2 summarizes the POS estimates (and expected cases) for each type for the results of the different scenarios presented in Figure 2 and Figure 3. The expected cases average over the variability that arises in the 100 stochastic iterations due to different risks of reintroduction. Table 2 divides the time period into pre-bOPV cessation (i.e., 2024–2027) and post-bOPV cessation (i.e., 2028–2035). The expected cases reflect estimates summed over multiple years and intended to provide context for relative comparisons for different policies, not precise estimates of future outcomes. As discussed in prior modeling, the expected annual case estimates depend on the modeled assumptions and time horizons, and they reflect substantial variability in the iterations [49]. These results suggest that intensification (i.e., increasing population immunity with additional bOPV pSIAs prior to bOPV cessation) could prevent substantial numbers of cVDPV1 cases during 2024–2027 compared with the baseline by increasing type 1 population immunity prior to the importation of cVDPV1 (or WPV1) from other countries or the development of cVDPV1 outbreaks in OPV-using countries. In addition, by adding more pSIAs in 2025 and 2026 with intensification, we reduced the competition between bOPV pSIAs and nOPV2 oSIAs in those years in some countries with co-circulation, which also led to few expected type 2 cases. The POS estimates in Table 2 show substantial improvement in the POS for both pre- and post-bOPV cessation for types 1 and 3 with bOPV pSIA intensification compared with the updated baseline with no intensification. Post-bOPV cessation, risks from the transition, iVDPVs, and containment breaches lead to some stochastic iterations, with some cases requiring outbreak response due to transmissions. The expected results in Figure 3 average over the 100 stochastic iterations, so the low numbers of expected cases for types 1 and 3 after 2028 reflect the averages of many iterations with 0 cases and some iterations with non-zero cases. Overall, as the POS increases, this reduces the expected number of cases, although for type 3, the expected annual case estimates remain below 100, and small numbers of iterations with outbreaks with intensification include a relatively higher number of cases because of their occurrence further out in time.

## 5. Discussion

Successful control of cVDPV1 outbreaks and outbreak prevention prior to bOPV cessation should keep the expected annual cVDPV1 cases relatively low prior to bOPV cessation, depending on pSIA scope, number, and quality. In contrast, low coverage of bOPV in RI and insufficient bOPV pSIAs to end all cVDPV1 transmission prior to cessation will necessitate rapid and high-quality oSIA response to prevent outbreaks from spreading internationally after bOPV cessation. Increasing type 1 and 3 population immunity using bOPV prior to cessation in pSIAs in many countries should represent a priority for countries and GPEI. Despite these improvements, the gradual expected increase in annual type 1 cases between 2029 and 2035, Figure 3A suggested that even with the modeled intensified bOPV pSIAs, bOPV cessation still represents an option with potentially unacceptable risk for type 1 (i.e., POS < 80%, Table 2).

The ongoing expansion of poliovirus vaccine options increases the complexity of planning for the polio endgame [69]. Discussions about the potential role(s) of nOPVs for type 1 and/or 3, potential multi-valent nOPV formulations (e.g., nbOPV, ntOPV), development of polio vaccines using non-replicating vaccine strains (e.g., vaccine-like particles [VLPs]), and/or different formulations of IPV (i.e., hexavalent combination products, fractional dosing of stand-alone IPV) make global modeling of policy and national decisions for immunization schedules and financing more difficult [69]. Countries now spend and should expect to spend, substantially more money on polio immunization than in prior years, at least in the absence of changes in immunization strategy [63,70]. In the absence of a successful global interruption of cVDPV2, restarting OPV2 in RI could become preferable to countries. GPEI promoted the preferential use of nOPV2 as an innovative tool to reduce the risks of seeding new VDPV2s [79]. Experience to date shows that while nOPV2 offers some reduction in VAPP and VDPV risks, prolonged shedding allowing recombination and reversion still occurs in the context of low-quality SIAs. Additionally, nOPV2 comes with real trade-offs of lower secondary spread compared with Sabin-strain mOPV2 [59]. Due to concerns about some increased potential for virus recombination as well as clinical trial results showing lower nOPV2 immunogenicity for individual vaccine recipients of co-administered nOPV2 and bOPV compared with those given nOPV2 alone [80], in 2023, SAGE recommended against co-administering nOPV2 and bOPV [81]. Complicated by co-circulation with type 1, we expect that type 2 transmission will likely continue throughout the time horizon, given current GPEI and national plans and the continued suboptimal implementation of oSIAs. In this analysis, we did not explore options for stopping type 2.

All applications of our integrated global modeling come with limitations due to assumptions about the model framing and structure, available information, initial conditions, and uncertainties about future policies and actions [28,48,49,58,59,60,61,62,63]. These results implicitly assume unlimited vaccine supplies for purposes of directly comparing policies; however, GPEI activities for cVDPV2 outbreaks continue to face challenges due to limited nOPV2 supplies, which could lead to substantially worse outcomes. Further, this analysis does not consider the potential impacts of increasing or decreasing coverage in RI and/or SIAs that may occur prospectively and could substantially shift outcomes. In addition, the model assumes a minimum of 1 dose of IPV in RI and does not include second IPV doses in RI in blocks that represent many countries that added these in response to recent SAGE recommendations [36]. However, while IPV use in RI helps to decrease the number of paralytic cases, it does little to increase the gastrointestinal immunity that is needed to reduce poliovirus transmission in OPV-using countries with conditions conducive to relatively higher transmissibility of polioviruses. The model includes IPV in some SIAs consistent with historical use but does not include the use of IPV in SIAs for oSIAs prospectively, except in IPV-only using countries. Although SAGE recently reinstated recommendations on the use of IPV in oSIAs [81], our modeling does not show IPV delivery in SIAs as effective or cost-effective for reducing cVDPV risks above OPV alone [82]. The effects of boosting existing immunity with IPV SIAs in children with prior live-poliovirus-induced immunity likely plays a minor role in stopping outbreak transmission because these children likely play a relatively less significant role in transmission than individuals in the population with no prior live-poliovirus-induced immunity [56,82]. Future global model updates will include the addition of the second dose of IPV, updated input immunization and population data, and the characterization of nOPV2 as a licensed vaccine.

The decentralization of global health decision-making, such as the preference of the African region to delay oSIAs in 2021 pending nOPV2 availability instead of using mOPV2, implicitly demonstrates a willingness to accept more polio cases as a trade-off [61], allows expansion of outbreaks, and complicates global policy modeling, planning, and implementation. Notably, the development of global resource needs (i.e., financial, vaccines) depends on assumptions about how decision-makers will act. When the modeling assumptions differ from actual decisions, this can result in mismatches between available and needed resources [19], more extensive transmission, and more cases [29]. Uncertainty about GPEI and country plans limits the ability of global modeling efforts to predict prospective outcomes. The expected values from these analyses do not consider adaptive adjustments in strategy that could occur in the event of increasing numbers of reported cases. For example, oSIA target age groups could increase to older ages to account for increasing numbers of birth cohorts without exposure to OPV immunization with increased years since OPV cessation (as recommended by pre-OPV2 cessation modeling [21]) and/or GPEI and countries could receive additional resources that could support interventions that perform better than modeled here.

We hope that this modeling will inform the development of future national and GPEI policies related to bOPV cessation. GPEI partners and public health leaders could use these results to provide a rationale for the development of a clear multi-year plan for the necessary financial and vaccine resources required to stop all WPV and cVDPV transmission (particularly established transmission of cVDPV2) and implement bOPV cessation in a way that will prevent a repetition of the experience of OPV2 cessation.

## 6. Conclusions

Our modeling shows that pSIAs with bOPV could substantially reduce the expected global risks of experiencing cVDPV1 and cVDPV3 outbreaks both before and after bOPV cessation and increase the probability of success for the polio endgame for types 1 and 3 but not for type 2. The decline and absence of bOPV pSIAs since 2019 in non-endemic countries and the postponement or cancellation of the small number of bOPV pSIAs into 2024 raises the risks of cVDPV emergence. In addition, unless GPEI makes explicit plans in 2024 to resume and continue bOPV pSIAs, efforts to ensure sufficient bOPV supply could be undermined as manufacturers could prematurely reduce production ahead of expected low demand [19].

## Figures and Tables

**Table 1:** Evolution of published estimates of the probability of success (POS) of OPV cessation.

Metric	Time Horizon [IC]	Type, POS ^a^ (Range)	Date(s) [Sources(s)]
Pre-OPV2 cessation studies (assumed OPV2 cessation would occur in 2016, bOPV cessation 2019)			
OPV restart	2013–2052 [End 2014 ^b^]	All types, 98% (90–98%)	24 Sep 2015 [23]
			24 Mar 2016 [21]
OPV restart	2013–2052 [End 2014 ^b^]	All types, 94.3% (15–94.3%)	23 Sep 2016 [64]
			1 Jun 2017 [65] (range)
Post-OPV2 cessation studies (OPV2 implemented in 2016; see text for assumptions for bOPV cessation)			
OPV restart	2019–2029 [End 2019]	Type 2, 11% (11–56%)	6 Jul 2020 [28]
OPV restart	2019–2029 [End 2019]	Type 2, 11% (33–78%)	10 Nov 2020 [59]
OPV restart	2019–2029 ^c^ [End 2019]	Type 2, 11%	19 Feb 2021 [70]
		Type 1; 2; 3 ^c^ 80%; 56%; 100%	
OPV restart	2019–2023 ^d^ [End 2019]	Type 2, 67% (62–73%)	27 Apr 2021 [60]
OPV restart	2019–2023 ^d^ [End 2020]	Type 2, 67% (46–67%)	14 May 2021 [61]
p(die out)	2022–2026 ^d^ [End 2021]	Type 2, <1% (<1–95%)	17 Nov 2022 [62]
p(die out)	2022–2035 [End 2021]	Type 1; 2 <1; <1%	21 Jun 2023 [48,49]
			14 Aug 2023 [73]

Abbreviations: bOPV, bivalent OPV; GPEI, Global Polio Eradication Initiative; IC, initial conditions; OPV, oral poliovirus vaccine; OPV2, type 2 OPV; p(die out), probability of die out of transmission by the end of the time horizon; POS, probability of successful OPV cessation; pSIA, preventive supplemental immunization activity; WPV1, type 1 wild poliovirus. ^a^ Column shows the poliovirus type(s) of POS estimates in the study and the expected POS for the modeled baseline (range of POS values for different scenarios considered, where applicable); see each study for specific details. ^b^ Model prospectively assumed GPEI and countries would implement all recommended OPV cessation risk management strategies. ^c^ For this study, baseline estimate POS of 11% for type 2, but hypothetical additional scenarios included pSIAs to achieve WPV1 eradication by 2023 and implemented bOPV cessation in 2025 with POS estimates for each type shown. ^d^ No consideration of bOPV cessation in this analysis since it would only potentially occur after the model time horizon.

**Table 2:** Probability of success (POS) estimates (expected total cases) for different time periods by type and scenario.

Scenario and Time Period	POS (Expected Cases) for 2024–2027						POS (Expected Cases) for 2028–2035					
Type		1		2		3		1		2		3
mOPV ^a^	0	(1151)	0	(19,004)	0.68	(100)	0	(164,443)	0	(84,645)	0.41	(200)
nOPV best ^a,b^	0	(932)	0	(6298)	0.81	(91)	0	(110,095)	0	(35,210)	0.41	(122)
nOPV worst ^a,b^	0	(1372)	0	(26,760)	0.74	(97)	0	(143,605)	0	(93,487)	0.41	(69)
Best nOPV, baseline ^b^	0	(5043)	0	(2558)	0.28	(120)	0	(45,571)	0	(7444)	0.41	(40)
Worst nOPV, baseline ^b^	0	(5043)	0	(19,146)	0	(125)	0	(105,401)	0	(62,558)	0.41	(103)
Best nOPV, intensification ^b,c^	0.97	(347)	0	(1661)	0.96	(96)	0.92	(1698)	0	(4797)	0.98	(18)
Worst nOPV, intensification ^b,c^	0.78	(382)	0	(14,895)	0.96	(87)	0.78	(10,100)	0	(57,103)	0.98	(70)

Abbreviations: mOPV, monovalent OPV; nOPV, novel OPV; OPV, oral poliovirus vaccine; POS, probability of successful OPV cessation by the date indicated; pSIA, preventive supplemental immunization activity; VAPP, vaccine-associated paralytic polio. ^a^ Results for the same time periods from prior bOPV cessation modeling that included assumed degradation of surveillance quality and resultant delayed outbreak response and did not include the use of tOPV in some countries where it occurred expanded age groups targeted in Yemen or any improvements in the quality of SIAs in the consequential geographies of DRC and Nigeria [48]. ^b^ Similar to prior modeling [48], we apply bounding scenarios of (i) *nOPV best*, which uses type-specific nOPV for outbreak response assuming the same effectiveness as type-specific mOPV, no reversion despite transmissibility, and no VAPP [59,78], and (ii) *nOPV worst*, which uses type-specific nOPV for outbreak response post-type-specific OPV cessation, assumes 90% of the effectiveness of mOPV and prior assumptions for reduced reversion [59], which we further reduced here by 10%, and VAPP occurring at a rate 10% lower than the VAPP rate of mOPV in vaccine recipients (see text). ^c^ Intensification refers to the addition of bOPV pSIAs prior to bOPV cessation in 2027 to some model subpopulations (see Figure 1 and text for discussion of the impacts of the planned rounds and the expected reductions of cases for types 1 and 2 relative to the baselines).

## Data Availability

The original contributions presented in the study are included in the article.
